# Arbitrary protein−protein docking targets biologically relevant interfaces

**DOI:** 10.1186/2046-1682-5-7

**Published:** 2012-05-06

**Authors:** Juliette Martin, Richard Lavery

**Affiliations:** 1 Université Lyon 1; CNRS, UMR 5086; Bases Moléculaires et Structurales des Systèmes Infectieux, IBCP, 7 passage du, Vercors, F-69367, France

**Keywords:** Protein structure, Protein-protein interaction, Docking, Interface prediction

## Abstract

**Background:**

Protein-protein recognition is of fundamental importance in the vast majority of biological processes. However, it has already been demonstrated that it is very hard to distinguish true complexes from false complexes in so-called cross-docking experiments, where binary protein complexes are separated and the isolated proteins are all docked against each other and scored. Does this result, at least in part, reflect a physical reality? False complexes could reflect possible nonspecific or weak associations.

**Results:**

In this paper, we investigate the twilight zone of protein-protein interactions, building on an interesting outcome of cross-docking experiments: false complexes seem to favor residues from the true interaction site, suggesting that randomly chosen partners dock in a non-random fashion on protein surfaces. Here, we carry out arbitrary docking of a non-redundant data set of 198 proteins, with more than 300 randomly chosen "probe" proteins. We investigate the tendency of arbitrary partners to aggregate at localized regions of the protein surfaces, the shape and compositional bias of the generated interfaces, and the potential of this property to predict biologically relevant binding sites. We show that the non-random localization of arbitrary partners after protein-protein docking is a generic feature of protein structures. The interfaces generated in this way are not systematically planar or curved, but tend to be closer than average to the center of the proteins. These results can be used to predict biological interfaces with an AUC value up to 0.69 alone, and 0.72 when used in combination with evolutionary information. An appropriate choice of random partners and number of docking models make this method computationally practical. It is also noted that nonspecific interfaces can point to alternate interaction sites in the case of proteins with multiple interfaces. We illustrate the usefulness of arbitrary docking using PEBP (Phosphatidylethanolamine binding protein), a kinase inhibitor with multiple partners.

**Conclusions:**

An approach using arbitrary docking, and based solely on physical properties, can successfully identify biologically pertinent protein interfaces.

## Background

It is now accepted as evident that protein-protein interactions (PPIs) are of fundamental importance in the vast majority of molecular events that occur in living organisms. Proteins can interact to form stable macromolecular assemblies that are able to perform many complex biological functions. They can also form transient interactions that collectively constitute dynamic networks of interactions that regulate how organisms operate. Protein-protein interactions are also of crucial importance to bacteria and viruses, which interfere with the host PPI network during infection [1,2]. Logically, protein-protein binding sites are becoming major targets for novel drug design strategies [3].

Shape complementarity, surface hydrophobicity and charge complementarity have all been recognized as key factors of recognition in early studies [4,5]. More recently, the increasing availability of structural data on protein-protein complexes [6] has led to a more refined picture of PPI mechanisms. Among the emerging structural and functional properties of transient interactions, one can cite conformational changes and disorder-to-order transitions upon interaction, the sequence conservation of interface residues, the existence of multi-specific proteins, and the role of post-translational modifications [7].

A number of methods for predicting PPIs have been developed, targeting two distinct aspects of the problem: protein-protein binding site prediction and protein docking. In the former case, the challenge is to identify the surface residues involved in the formation of protein-protein complexes; see [8-10] for recent reviews. In contrast, docking methods aim at predicting the structures of known, generally binary, complexes starting with the structures of separate proteins and using scoring functions based on shape/electrostatic/hydrophobic factors to locate optimal conformations. Substantial progress has been made in the docking field over recent years. The best algorithms are now able to predict correctly the structures of most complexes, when no major conformational change occurs during interaction, and promising developments are being made in the treatment of conformational changes [11,12]. It has however been pointed out that the scoring functions used in docking perform very poorly when the aim is to predict binding affinities [13-15]. Notably, "cross-docking" studies, where binary protein complexes are separated and the isolated proteins are all docked against each other using a successful multiple minimization docking algorithm [15,16], have demonstrated that it is very hard to distinguish between "true" (native) and "false" complexes. Similar difficulties were found using the top-performing Cluspro [17] web server [J. Martin unpublished results]. In another study, carried out on a larger scale, and using another docking algorithm [18], despite docking scores biased in favor of true complexes, the vast majority of cases led to false complexes being scored better than true ones.

The fact that false complexes obtain good scores during cross-docking studies raises two important and orthogonal questions: Are scoring functions so poor that they cannot discriminate interacting from non-interacting proteins (as suggested by the observations of [13,14]), or does this result, at least in part, reflect a physical reality? Unfortunately, there is virtually no experimental data on the strength of the interactions comprising the "false" complexes. This set of complexes could potentially reflect potential weak, or nonspecific, interactions that are present in the cytoplasm, or avoided by mechanisms such as compartmentalization.

The fact that biological interactions in the cell are tightly orchestrated by localization and co-regulation mechanisms indeed suggests that significant nonspecific interactions may be common. It has been proposed that co-localization is necessary to control specific interactions, given the size of cells and the lifetime of individual proteins [19]. So far, nonspecific interactions have only been marginally addressed in the literature, but they certainly deserve more attention. If localization and co-regulation is the rule in healthy cells, singular events also occur where localization breaks down, for example when mitochondrial proteins are released into the cytoplasm during the early phase of apoptosis, or when viral or bacterial proteins interfere with the host PPIs during infection. Recent studies indeed suggest that weak interactions play an important role in complex systems. A pioneering simulation of the bacterial cytoplasm has shown that proteins interacting with hard-sphere potentials diffuse too fast compared to experiment and that adding weak nonspecific attractions between all proteins could correct this behavior [20]. Another recent study suggests that nonspecific binding acts as the evolutionary level to shape the PPI networks and limits the number of different proteins in genomes [21]. Ultimately, a full understanding of proteins networks can only be achieved if we address the nonspecific as well as the specific interactions.

In this paper, we investigate what can be termed the "twilight zone" of protein-protein interactions, by using computational docking, and building on an interesting outcome of earlier cross-docking experiments: "false" complexes seem to favor interfaces containing residues belonging to "true" interaction sites [15,18]. This suggests that randomly chosen protein partners dock in a non-random fashion. Using a non-redundant data set of 198 proteins, we explore the tendency of randomly chosen partners to aggregate at localized regions on the surface of each protein. We analyze the shape and compositional bias of the interfaces that are generated and the potential of this approach for predicting biologically relevant protein binding sites. We test our procedure on PEBP (Phosphatidylethanolamine binding protein), a kinase inhibitor with multiple known partners.

## Methods

### Data sets

We have extracted protein structures from the docking benchmark data set assembled by Hwang et al., version 4.0 [22]. This set consists of 176 binary protein complexes, for which structures of both partners in bound and unbound forms are available. These complexes are classified into functional categories (enzyme/inhibitor, antibody/antigen and "other") and according to the probable challenge for docking algorithms, which is related to conformational change upon complex formation (rigid body, medium difficulty and difficult). We first excluded the complexes from the antibody-antigen category (which do not display conventional PPI interfaces), and then reduced the redundancy level to 30% between protein chains using the PISCES web server [23]. The final data set, denoted hereafter as the *target* data set, encompasses 198 proteins. Only unbound forms were used in all docking experiments. The structures were downloaded from http://zlab.umassmed.edu/benchmark/benchmark4.tgz.

These 198 proteins were docked against arbitrary partners taken from Nh3D, a data set of representative structures of each domain at the Topology level of the CATH structural classification database [24]. Starting from version 3.0 of this data set (806 structures), we removed proteins with gaps in their backbone coordinates and proteins with high radius of gyration compared to their length (see Additional File 1).

To remove known structural interactions between arbitrary docking partners and the proteins from the target set, proteins classified in the same CATH Topology level as proteins of the benchmark 4.0 were removed. The final set includes 314 structures. It contains respectively 2, 91, 105, 53, 36 and 13 chains in length brackets ranging from 0–50 up to 250–300, and 14 chains with more than 300 residues. This data set is denoted as the *compact probe* data set. We further checked for known interactions between these probes and the target proteins using the IntAct database [25] and found only 16 interactions (see Additional File 1).

To investigate the role of structure compactness, we also considered a data set of 20 partners having a high radius of gyration compared to their length (see Additional File 1), denoted as the *extended probe* data set.

### Docking

Docking was performed with the Hex software [26], version 6.3, which is adapted to GPU processors. Computations used the shape complementarity scoring function, with 18 and 25 expansion orders for the initial and final search steps (these orders control the precision of the molecular representation and influence the computation time). The full list of parameters is given in Additional File 1.Unless otherwise stated, we used only the best conformation of the complex produced by Hex.

### Analysis of docking results

Accessible surface areas were computed using NACCES [27]. Exposed residues were defined as those with a relative accessible surface area (RSA) greater than 5%. Interacting residues were defined as those with heavy atoms less than 5 Å away from heavy atoms of the interacting protein. Following docking with the set of arbitrary partners, we counted the *number of docking hits* for each exposed residue, that is, the number of times that a residue is seen in interaction with a docking partner. To allow comparison between proteins of different size, the number of docking hits per residue was normalized using the formula:

(1) $$N_{\mathrm{norm}}=\frac{N-min\left(N\right)}{max\left(N\right)-min\left(N\right)}$$

where *min*( *N*) and *max*( *N*) denote the minimum and maximum number of hits observed for each protein. Using this normalization, the number of hits per residue lies in the range [0,1].

The link between docking hits and surface shape was investigated using: (i) a local planarity analysis; (ii) the relative closeness to the geometrical center of the protein. The local planarity was measured with a planarity index, P_IND_, computed for each exposed residue as follows. Each residue was taken as the seed of a local surface patch, including all atoms of neighboring exposed residues within a 10 Å radius. To avoid discontinuities of these local surface patches (when for example, the radius selection includes residues from both sides of the protein), residues were filtered using hierarchical clustering with a single linkage procedure; the resulting tree was truncated using an empirical cutoff of 4.2 and secondary clusters were removed (see Additional File 1). P_IND_ was then defined as the root mean squared distance of all atoms from the mean least squares plane [28]. A low P_IND_ denotes planar patches, while a high P_IND_ denotes curved patches (pockets and protrusions). Each residue was also associated with a patch score, which is the mean number of normalized docking hits occurring in the patch around this residue.

To determine the closeness of an exposed residue to the geometric center (GEOCEN) of a protein, we built on the definition of Nicola and Vakser [29], by first reducing the protein to its Cα atoms. The relative closeness (R_GEOCEN_) of an exposed residue is then the ratio between its distance to the GEOCEN, and the mean distance of all exposed residues to the GEOCEN. A value below 1 consequently indicates that a residue is closer than average to the GEOCEN.

### Statistical testing

All statistical tests were performed using R [30]. Docking hit distributions and random hit distributions were compared using the Cramer test, implemented in the Cramer package [31]. We used this test rather than the Kolmogorov-Smirnov test because it can handle discrete distributions with ties [32].

The correlation between the number of docking hits and the patch planarity was measured using Pearson’s product moment correlation coefficient. Since the distributions we observe are not all Gaussian, we computed empirical p-values with 1000 replicates.

To take into account multiple testing, p-values were systematically corrected using the Benjamini-Hochberg method (BH) [33]. Unlike the classical Bonferroni correction, the BH method controls the false discovery rate (proportion of significant cases that are false positives). With a risk level set to 5%, the probability to get at least one false discovery is 5% using the Bonferroni correction, whereas with the BH correction, the expected percentage of false discovery is 5%. Since we were more interested in the global number of significant p-values than the individual analysis of significant cases, we preferred the less stringent BH correction.

The amino-acid composition bias in regions with many docking hits was assessed using the Chi-squared test. Significant contributions to Chi-squared were detected by looking at the Pearson residuals: $\frac{X_{\mathrm{observed}}-X_{\mathrm{expected}}}{X_{\mathrm{expected}}}$, which, by construction, follow a *N*(0,1) distribution. The Chi-squared test is a test of independence between variables. It determines whether or not the variables are independent, but, when independence is rejected, it does not quantify the association between the variables. To get a quantitative measure of the association, we considered the Cramer coefficient, Cramer’s V, defined by:

(2) $$V=\sqrt{\frac{Chi^{2}}{Chi_{\max}^{2}}}$$

Where $Chi^{2}$ denotes the Chi-squared value obtained for the contingency table, and $Chi_{\max}^{2}$ denotes the theoretical maximal Chi-squared of the contingency table, given by $Chi_{\max}^{2}=Nxmin\left(r-1,c-1\right)$ where *N* is the total count of the table and *r* and *c* are the table dimensions. Cramer’s  *V* is, by construction, always between 0 (independence) and 1 (full dependence) and has a clear proportional interpretation: it is the fraction of the maximal departure from independence that one would observe in case of full dependence [34].

The difference between AUC values (see “Prediction assessment”) was assessed using the non-parametric DeLong’s test [35] implemented in the pROC package [36].

### Prediction assessment

Biological interfaces were defined using the bound structures of the docking data set version 4.0 [22] with a 5 Å distance cutoff between heavy atoms.

The performance in prediction was assessed using the False Positive Rate (FPR) and True Positive Rate (TPR) defined by $FPR=\frac{FP}{FP+TN}$ and $TPR=\frac{TP}{TP+FN}$, where *FP* denotes the number of false positives (residues incorrectly predicted as interfacial), *TN* denotes the number of true negatives (residues outside the interface and rejected by the prediction), *TP* denotes the number of true positives (interface residues predicted as such) and *FN* denotes the number of false negatives (interface residues missed by the prediction). The computation of FPR and TPR for various thresholds enables a receiver operating characteristic (ROC) curve to be drawn. A unique measure of performance is then given by the resulting AUC value (area under the curve) that is equal to 0.5 for a random prediction and 1.0 for a perfect prediction. The prediction was carried out using exposed residues (RSA > 5%). AUC values were computed using the pROC package [36].

### Comparison with other methods

VORFFIP is a recent and sophisticated method for predicting protein interaction surfaces which uses different forms of information including structural features, energy terms, evolutionary conservation and crystallographic B-factors, with a Voronoi-based environment description, combined in a two-step random forest classifier [37]. The authors of this method kindly provided VORFFIP scores for the proteins in our target set.

JET is a method based on evolutionary trace [38]. It uses a sampling of distance trees, and a post-processing clustering that takes into account physico-chemical properties as well as the evolutionary trace of the residues. We used the iterative version of JET with 10 iterations, and used the number of iterations where a given residue appeared in a cluster as a predictor (column ’clustersOccur’ of the _jet.res files).

### Detection of multiple interfaces

PiQSi is a curated database of protein quaternary structures [39]. Structures stored in PiQSi are manually examined and annotated as correct or incorrect, and it is possible to retrieve homologues of a given protein. Around 15% of the structures are annotated as erroneous. This resource was used to retrieve homologues of the target proteins involved in protein complexes and detect multiple interfaces. Structures were superimposed using PyMOL [40], which was also used to generate images of structures.

## Results

### Docking hits target localized regions of protein surfaces

**Figure 1:**
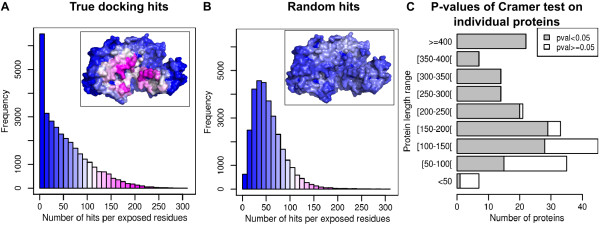
**Distribution of docking hits on the protein surface is not random.A**: global distribution of hits generated by docking the 198 proteins in the target set with the 314 compact probes. **B**: global distribution of hits generated with a random model (see text). The insets illustrate the hit distributions on the surface of human HLA class 2 histocompatibility antigen (PDB code: 1 H15, chains A and B [41], the unbound form of 1KLU_r [42]). **C**: analysis of individual proteins. Cramer's test is used to compare the docking and random hit distributions for each protein. Grey: significant p-values, white: non-significant p-values. The significance level is set to 5% and p-values are adjusted using the Benjamini-Hochberg method.

We first study the distribution of docking hits on the surface of the 198 target proteins after docking with the 314 compact probes. For each exposed residue, the number of times it belongs to an interface is recorded. Global results are given in Figure 1A. For comparison, the distribution of hits expected using a random model is shown on Figure 1B. With the random model, a surfaces patch is created by randomly choosing an exposed residue and generating a patch of the desired size by integrating its nearest neighbors. To ensure patch continuity, a residue is integrated into a patch only if it is sufficiently close to residues already in the patch (inter-Cα distance lower than 3.8 Å; this cutoff is gradually relaxed if no residue satisfies the constraint). The sizes of random patches are set equal to the sizes of the interfaces generated by the arbitrary docking experiments. As can be seen on Figure 1A, the number of hits per residue generated by docking experiments shows an exponential decrease, with most residues having no, or few, hits and a small number of residues with many hits. This reflects highly localized interaction regions, as illustrated in the inset showing a typical protein. This result is very different from the random distribution shown in Figure 1B, which has a negative binomial shape, with a peak around 50 hits.

Extending the global analysis to individual proteins, we compute the distributions of observed and expected number of hits separately for each protein, and compare them with the Cramer test. The target proteins are binned in terms of their length range, and the number of significant p-values in each bin is shown in Figure 1C. For a total of 198 proteins, 150 have a significant p-value (<0.05), meaning that the distribution of docking hits on their surface significantly differs from random. There is a clear influence of protein size: shorter proteins tend to have non-significant p-values (implying random hits). A possible explanation is that smaller proteins get “saturated” faster than larger ones during docking with probes of various sizes. In other words, it is more difficult to target a precise site on the surface of a small protein using partners of diverse sizes. When docked with large partners, a significant portion of the surface will automatically be contacted; this suggests that small docking probes might be better adapted to detecting interaction interfaces on small proteins. To test this hypothesis, we divided the set of compact probes in half based on their size: we obtained 159 significant p-values out of 198 with the shortest probes, but only 133 significant p-values with the longest probes (versus 142 with half of the compact probes randomly chosen). This supports our hypothesis that small docking partners yield more significant p-values, and can detect localized interactions regions better, even on small protein targets.

The data shown on Figure 1 illustrate the first major result of this study: large-scale docking with a set of random partners, chosen to reflect the diversity of protein folds, reveals highly localized interaction regions on the surface of most of the target proteins. This is true both globally and individually for the set of 198 target proteins. These highly localized zones are termed “favored regions” and are the subject of further analysis below.

### Docking hits do not accumulate on planar sites, or in pockets, but are generally close to the center of proteins

In this section, we explore the features of favored regions in terms of protein shape, using measures of local planarity and closeness to the geometrical center of the target proteins.

The local planarity around each exposed residue of the 198 target proteins is measured with the P_IND_ planarity index described in the Methods section. The P_IND_ of each residue is then compared with the corresponding patch score, which is the mean number of docking hits per residue in the local patch generated around the residue. To allow comparison among proteins of different size, the number of hits per exposed residue has to be normalized for each protein as explained in the Methods section. The global correlation between planarity index and patch score is very weak, with a Pearson correlation coefficient rho equal to −0.026 (empirical p-value < 0.001), implying that there is no link between local planarity and accumulated docking hits (see Additional File 1). This result is unchanged when the size of the local patches generated to measure the P_IND_ is increased (rho = −0.057 with a distance cutoff equal to 15 Å).

**Figure 2:**
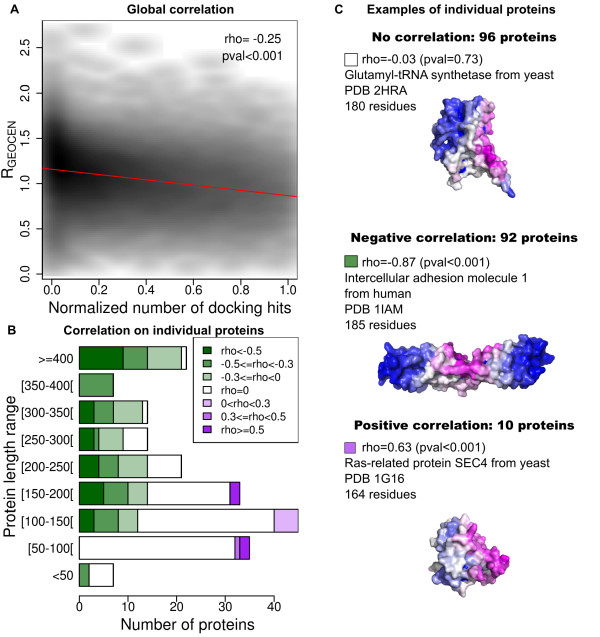
**Docking hits tend to accumulate near the center of proteins.A**: global correlation between the relative distance to the geometric center of the protein, R_GEOCEN_, and the normalized number of docking hits, for exposed residues within the target set of 198 proteins, after docking with the 314 compact probes. The Pearson correlation coefficient and associated empirical p-value are shown on the graph. The regression line, *y*=1.16-0.29x, is shown in red. **B**: individual correlations for the 198 proteins of the target set. **C**: illustrative examples of proteins with zero, negative, and positive correlation. No correlation: 2HRA [43] (unbound form of protein 2HRK_r [44]). Negative correlation: 1IAM [45] (unbound form of protein 1MQ8_r [46]), docking hits concentrate near the geometric center. Positive correlation: 1 G16 [47] (unbound form of protein 3CPH_l [48]), docking hits concentrate on a protrusion, far from the center.

The R_GEOCEN_ index described in the Methods section quantifies the proximity of an exposed residue to the geometrical center of a protein compared to the average distance of exposed residues to the geometrical center. The comparison of R_GEOCEN_ with the normalized number of docking hits per exposed residue reveals a global trend: the correlation is significantly negative, with a rho coefficient equal to −0.25, see Figure 2A. Thus docking hits tend to accumulate closer to the geometrical center than average. The correlation analysis of individual proteins reveals that while 96 proteins show no correlation (empirical p-value > = 0.05), 102 proteins display significant correlation, of which 92 are negative, see Figure 2B. Figure 2C illustrates the three possible cases of correlation. Using an arbitrary cutoff of rho = 0.3, 60 proteins remain negatively correlated and 5 positively correlated. The correlations show a link with protein chain length as very few correlations are found for short proteins. We also detected a significant link between the rho coefficients and the dispersion of R_GEOCEN_ for each protein: negative rho corresponds to proteins with a high dispersion of the relative distances, that is, conformationally anisotropic proteins (See Additional file 1). The fact that the docking is based on shape complementarity favors the formation of large interfaces, which are probably easier to achieve with more anisotropic proteins.

Taken together, the result of local planarity analysis and the data presented in Figures 2 illustrate our second major finding: favored regions do not occur systematically on planar sites, or in pockets, or on protrusions, but do tend to prefer residues closer to the center of the protein than average (a feature previously demonstrated for the interfaces of known complexes [29]).

### Docking hits prefer hydrophilic residues

**Figure 3:**
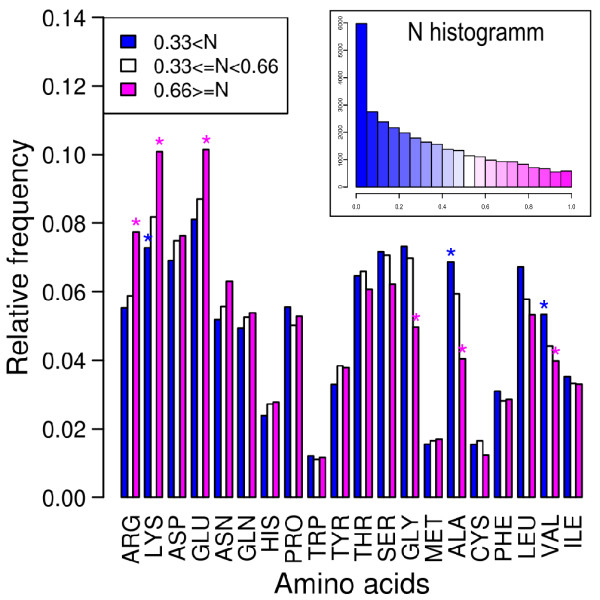
**Favored regions are enriched in charged and hydrophilic residues.** Amino-acid composition as a function of *N*, the normalized number of docking hits, for all exposed residues in the target set of 198 proteins after docking with the 314 compact probes. Stars show the cases that make significant a contribution to the Chi-squared test. The right corner inset shows the global distribution of the normalized number of docking hits.

We now investigate the amino-acid composition of favored regions. Surface residues are partitioned into three bins, according to the number of normalized docking hits, and we compute the amino-acid frequency in each bin. The result of this analysis is given in Figure 3. We find a significant coupling between the number of docking hits and the amino-acid composition, as assessed by the Chi-squared test (p-value < 2.2e-16). Regions with a high normalized number of hits are enriched in charged and hydrophilic residues (arginine, lysine, glutamate) and depleted in hydrophobic residues (alanine, leucine), as well as glycine. However, Cramer's V coefficient shows that this bias is very weak: V = 0.06, meaning that there is only a 6% departure from independence. Note that the correlation cannot be explained by a bias of the docking procedure, which relies only on shape complementarity between partners, and, notably, does not treat electrostatics.

This is the third result of our study: favored regions are weakly enriched in charged or hydrophilic residues.

### Can arbitrary docking help to predict specific interfaces?

The logical extension of our finding is the use of the favored regions, generated by docking with random partners, to predict the location of native interfaces. The fact that false complexes can help to identify correct interfaces was noted in the first cross-docking experiment on twelve proteins [15], and visually assessed for ten proteins in the study of Wass et al. [18]. Here, we quantify the predictive power of arbitrary docking on a larger data set, and assess its practical applications.

**Figure 4:**
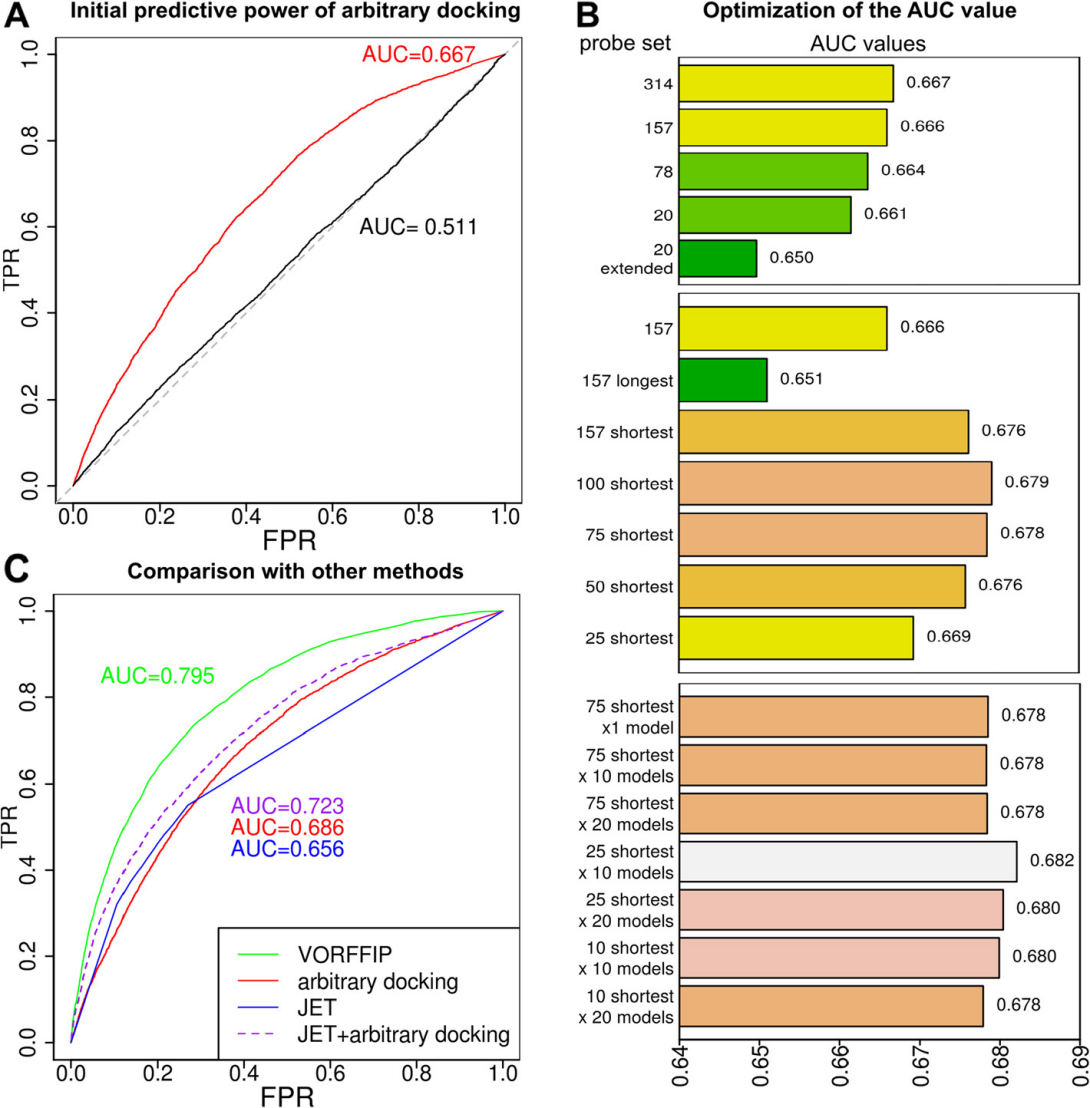
**Arbitrary docking has the power to predict native interfaces.A**: Initial ROC curves obtained for all exposed residues in the target set of 198 proteins. In red, the number of docking hits after docking with the 314 compact probes is used as a predictor. In black, for comparison, the ROC curve obtained using hits generated with the random model shown in Figure 1B. The number of hits per exposed residue is normalized for each protein as described in the Method section. **B**: Optimization of the number and size of the probes, and the number of docking models per probe, to increase the AUC value. Top: varying the number of probes. Middle: varying the size of the compact probes. Bottom: varying the number of docking solutions. Bars with the same color correspond to AUC values that are statistically indistinguishable (p-val > 5%). **C**: Comparison with other methods, considering predictions obtained by arbitrary docking with the 25 shortest probes and the first 10 docking models. The combination of arbitrary docking hits with JET is detailed in the text.

The performance of the normalized number of docking hits as an interface predictor is shown in Figure 4. Figure 4A presents ROC curves obtained using the actual docking hits, compared to random hits, whose distributions were shown in Figure 1, after normalization. It can be seen that the normalized number of docking hits yields significant predictive information, with an AUC value equal to 0.667, versus 0.511 for random hits. A purely physical index, based on shape complementarity with arbitrary partners, thus does contain a predictive signal. We can relate this finding to the compositional bias identified in the preceding paragraph: favored regions were found to be enriched in arginine, lysine and glutamate, and depleted in alanine, leucine and glycine. In our data set, we found an enrichment of arginine, lysine and glutamate, and a depletion of alanine and leucine at the periphery of interfaces (the “rim” regions, see Additional file 1).

However, carrying out arbitrary docking against 314 random partners, even accelerated on GPUs, is a time-consuming procedure. To be usable in practice, it is desirable to find a way to reduce the computation time. In Figure 4B, we show that we can vary the number and size of the probe protein set, as well as the number of docking models, to decrease the amount of computation without damaging performance. We first tested reducing the number of probes used: the predictive power (assessed by the AUC) is not affected if we use only half of the compact probes randomly chosen, with an AUC equal to 0.666, but does decrease with further reductions. We also found that extended probes perform worse than compact ones (see Figure 4B): AUC is equal to 0.661 with 20 compact probes versus 0.650 with 20 extended probes. In terms of protein size, we found that when sorting the probes into two equal sets based on chain length, shorter chains function better (AUC is equal to 0.676 with short probes versus 0.651 with long probes) in agreement with the observation that smaller proteins can locate interfaces more precisely. Encouraged by this finding, we again decreased the size of the probe data set, but this time always keeping the shortest probes. In this case, the predictive power remained intact with as few as 75 probes, with an AUC equal to 0.678.

Lastly, we studied a third parameter: the number of docking models analyzed per probe. We found that the best performance (AUC = 0.682) is achieved using the first 10 models and only the 25 shortest probes. This suggests that arbitrary docking could be used in practical applications, since predictive power can be obtained with a very limited number of docking computations.

We now consider the performance of this approach compared with other existing methods (see Figure 4C). The first method we compared is VORFFIP [37]. This method achieves an AUC equal to 0.795 on the target data set, whereas arbitrary docking, using 25 shortest probes and 10 models, achieved an AUC equal to 0.686. Since our method is based on only one feature, namely the information provided by arbitrary docking, we did not expect to equal the performance of a highly sophisticated multi-term method such as VORFFIP, which, today, can be considered to represent an upper bound on predictive power.

Next, we compared our performance to JET, which is based on sequence information, with a post-processing clustering [38]. Using JET results, we achieved an AUC equal to 0.656. Since JET and arbitrary docking are based on two orthogonal sets of data (evolutionary versus physical), it seemed interesting to test a combination of the two predictors. A simple linear combination, with a weight equal to 0.6 for arbitrary docking and 0.4 for JET, led to an increase in AUC to 0.723. This very encouraging results shows that two features, conservation and arbitrary docking, can make very good predictions.

The fourth message of our study is therefore that arbitrary docking is computationally practical (with an appropriate choice of probes and docking models) and either alone, or combined with other data, provides significant information for predicting biologically-relevant protein interfaces.

### Arbitrary docking can point to alternate interfaces

**Figure 5:**
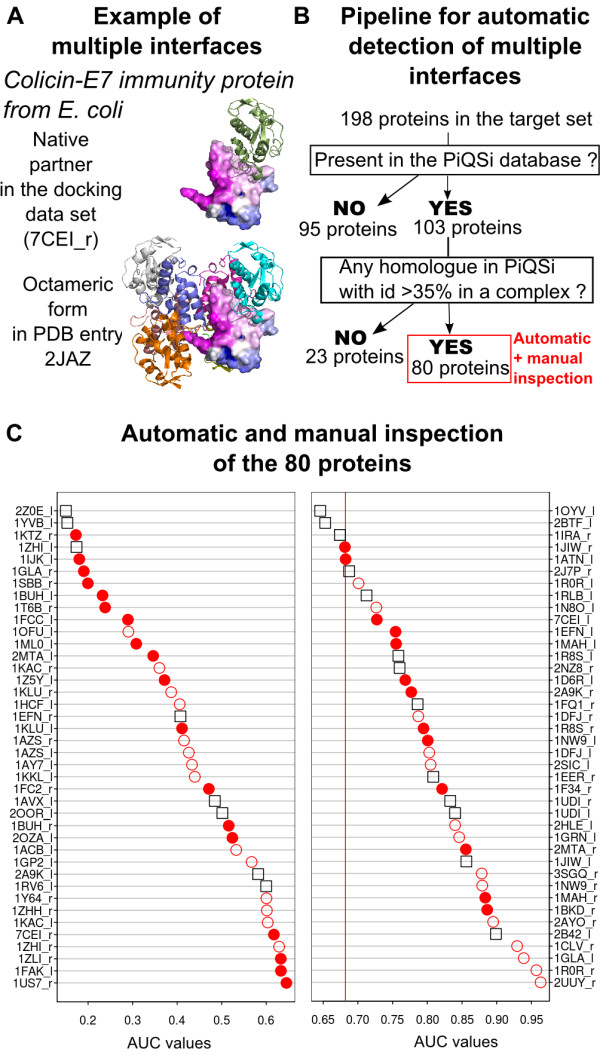
**Arbitrary docking also targets alternative interfaces.A**: Example of a protein for which the prediction of the biological interface from arbitrary docking appears to fail. The Colicin-E7 immunity protein forms a dimer with the E7 protein in the bound structure of the docking data set (7CEI_r [49], unbound form 1UNK [51]). This protein also forms an octamer involving four Colicin-E7 immunity proteins and four E7 proteins in structure 2JAZ [50]. The surface of Colicin-E7 immunity protein is colored according to the number of docking hits obtained with the 25 shortest probes and 10 docking models, highlighting the fact that arbitrary docking targets a region involved in the interface of the octamer. **B**: Description of the procedure used to detect multiple interfaces with the PiQSi database. Starting from the 198 proteins in the target set, we obtain 80 proteins for which PiQSi retrieves homologues with more than 35% identity involved in protein complexes. These 80 proteins are subjected to further inspection as described in the text. **C**: Result of the inspection of the 80 proteins. Proteins are ranked according to the AUC values returned by the arbitrary docking using 25 shortest probes and 10 docking models. Black squares: proteins for which the automatic inspection reveals no multiple interfaces. Red circles: proteins for which the automatic inspection reveals multiple interfaces. Filled circles: visual inspection reveals that arbitrary docking targets a region involved in other interfaces, as exemplified in panel A, open circles imply that visual inspection does not reveal such trend. The vertical red line represents the global AUC obtained for the target data set.

Although the predictive power of arbitrary docking itself is significant, some proteins seem very difficult to treat. Further examination of the difficult cases led to interesting cases of proteins that probably have multiple interaction interfaces. The apparent failure of arbitrary docking can indeed result from detecting interfaces that exist in alternate complexed forms of a protein, distinct from those described in the docking benchmark data set. Figure 5A shows one such example for the Colicin-E7 immunity protein, which is involved in a binary complex with the E7 protein in the docking benchmark data set, corresponding to the PDB structure 7CEI [49]. Arbitrary docking targets a region outside the 7CEI interface, resulting in a low predictive signal (AUC = 0.62). This protein is however also present in the PDB in an octamer, formed by four Colicin-E7 immunity proteins and four E7 proteins, in structure 2JAZ [50] (the quaternary structure was assigned by authors) and the region highlighted by arbitrary docking is indeed involved in the region highlighted by arbitrary docking.

To extend this type of analysis to all the proteins of the target data set, we developed the procedure outlined in Figure 5B. We first used the PiQSi search engine to retrieve homologues of our proteins with more than 35% identity. We then restricted the set of homologues to the ones involved in protein complexes. At this stage, we excluded complexes annotated as errors or probable errors, but retained structures that are not yet annotated, which is the case for most of the proteins retrieved. This yielded a restricted set of 80 proteins that were subjected to further automatic and manual examination. We first automatically detected multiple interfaces: retrieved homologues were superimposed on the target proteins using the mapping information provided by PiQSi and interfaces were extracted using a 5 Å distance cutoff. A multiple interface was detected if more than 10 residues not belonging the native interface (the one present in the docking data set) were involved in another interface. In this way, we detected 59 proteins out of 80 as having multiple interfaces. These 59 proteins were visually inspected to see if arbitrary docking targeted the new interfaces. We found 31 such cases out of 59. The results are presented in Figure 5C, where proteins are ranked according to the quality of the predictions of the native interface. For the 45 proteins with a predictive signal lower than average, 20 cases with multiple interfaces are observed. This implies that the predictive power is actually underestimated for at least 20 of these proteins.

The data presented in Figure 5 illustrates the last message of our study: apparent failures of arbitrary docking in predicting biologically relevant interfaces may indicate alternative protein interaction sites.

### A test case: PEBP

We conclude this study by showing the insight arbitrary docking can provide with a test case: the protein PEBP (Phosphatidylethanolamine binding protein). This protein, also called RKIP (Raf Kinase Inhibitor Protein), is a known inhibitor of several protein kinases, including those of the Raf/MEK/ERK pathways and the Aurora B pathway. PEBP also inhibits the GRK2 kinase, when activated by protein kinase C. These functionalities are the result of direct physical interactions, although no structural details of the complexes exist (personal communication from F. Schoentgen). Nevertheless, the structures of PEBP and some of its partners are available in the PDB. We thus subjected PEBP to arbitrary docking to highlight putative interaction sites, and also docked PEBP with its known partners using the Clus-Pro web server [17]. We considered the following protein structures: human PEBP (PDB code 1BD9 [52], chain A), human B-Raf kinase (PDB code 3PRF [53]), human MEK1 (PDB code 3E8N [54]), human ERK1 (PDB code 2ZOQ [55], chain A), human TAK1 kinase-TAB1 fusion protein (PDB code 2EVA [56]), Xenopus aurora kinase (PDB code 2VGO [57], chain A), human protein kinase C-beta II (PDB code 2I0E [58], chain A) and human GRK2 (PDB code 3CIK, chain A). All these proteins are known to be functional as monomers (personal communication from F. Schoentgen), and we thus docked only monomeric forms, even if the biological unit from the PDB was not monomeric.

**Figure 6:**
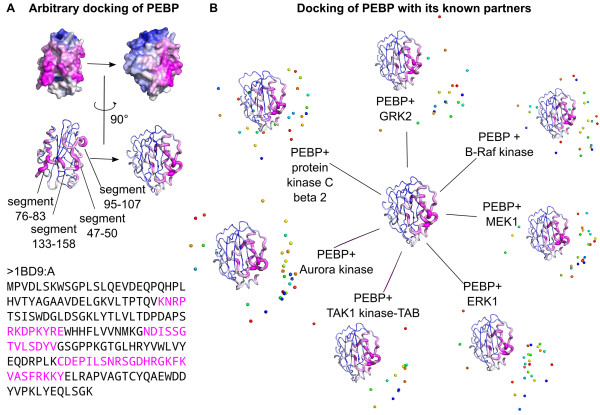
**Test case on PEBP.A**: Result of arbitrary docking of PEBP (PDB code 1BD9) with the 25 shortest random partners, using 10 docking models. The structure of PEBP is colored according to the number of hits; in the lower part, the thickness of the cartoon representation is proportional to the number of hits. **B**: Results of docking PEBP with its known partners using ClusPro. Each partner is represented by its centroid; different docking solutions are indicated by different colors.

The results of this study are illustrated in Figure 6. In Figure 6A, we show the result of arbitrary docking of PEBP with 25 random partners and using 10 docking models. It can be seen that docking hits clearly concentrate on one side of the protein. The preferred region encompasses four non-contiguous segments: regions spanning residues 47–50, 76–83, 95–107 and 133–158. Interestingly, regions 76–83 and 133–158 are known to be involved in the binding of anionic ligands [59], and helix 151–158 is phosphorylated by PKC and is thus involved in physical interaction with this kinase. Figure 6B summarizes the results of docking PEBP with its known partners using ClusPro. In each case, the shortest protein chain (PEBP) was used as ligand and the longer one as receptor. We considered all the models produced by ClusPro using the scoring function termed « balanced ». It is striking to note that for each known partner, there is a tendency to dock on the zone detected by arbitrary docking. This illustrates the practical use of arbitrary docking and suggests that, in the case of PEBP, diverse partners probably interact at the same interface.

Lastly, it is interesting to contrast this study with other related research on protein binding sites. Here, we addressed a specific question: do computational docking experiments applied to random protein partners lead to specific bound conformations? We found that, generally, such conformations are not random and the interactions tend to favor specific sites on the protein surfaces. A similar behavior is observed for interactions between proteins and small molecules, both in vitro and *in silico*. In vitro, the multiple solvent crystal structures (MSCS) method involves solving the X-ray structure of a protein in different organic solvents. The solvent molecules effectively probe the protein surface and tend to form clusters at protein binding sites [60]. *In silico*, the FTMAP algorithm has been developed to perform a fast Fourier surface mapping using the rigid-body docking of 16 small molecules with a given target protein [61].

Another notable feature of protein-protein interfaces is the uneven contribution of interface residues to the binding free energy. Generally, only a few residues, termed hot spots, make major contribution. Hot spot residues are enriched in tryptophan, tyrosine and arginine, but depleted in leucine, threonine and valine. They are preferentially located towards the center of the protein interface and appear in clusters [62-64]. There are, however, a number of protein-protein complexes that seem to be devoid of hot spots, and the hot spot nature of a residue may also change as a function of the surrounding protein interface [64]. In the present study, we did not address the question of hot spots directly, but we did observe a consistent compositional bias favoring arginine and valine residues in regions targeted by docking hits. Further studies will be necessary to determine if hot spots make a significant contribution to the preferred protein complex conformations we have found.

## Conclusions

We have shown that docking target proteins against an arbitrary set of proteins, leads to a non-random localization of interaction interfaces. These interfaces are neither systematically planar nor curved, but do tend to be closer than average to the center the protein. These predicted interfaces have been shown to contain information on the location of functional, biological interfaces, including alternative interfaces with multiple partners. An appropriate choice of random partners and of docking models analyzed makes arbitrary docking a practical tool for interface detection. The results can be used alone, or can complement data coming from other sources.

## Competing interests

The authors declare that they have no competing interests.

## Authors’ contributions

JM participated in the design of the study, performed the statistical analysis and drafted the paper. RL participated in the design of the study and helped to draft the manuscript. Both authors read and approved the final manuscript.

## Supplementary Material

Additional file 1: Supporting Information This file contains: Additional file 1 figure  S1 showing the features of the probes selected from the Nh3D data set (radius of gyration versus size), Table S1 showing the interactions between the 198 targets and the 314 random partners found in the Intact databank, Table S2 showing HEX parameters, Additional file 1 figure  S2 illustrating the procedure of local patch generation for P_IND_ computation, Additional file 1 figure  S3 showing the lack of correlation between P_IND_ and accumulated docking hits, Additional file 1 figure  S4 showing the rho coefficients of Figure 2 versus standard deviation of R_GEOCEN_, the relative distance to the geometric protein centers, Additional file 1 figure  S5, showing the multiple interfaces of the 31 proteins detected in Figure 5 and Additional file 1 Figure  S6, showing the composition of surface/interface rim/interface core regions in the target data set. Click here for file
