# Supplementary material for: Arbitrary protein−protein docking targets biologically relevant interfaces
Source: BMC Biophys. 2012 May 6;5:7. doi: 10.1186/2046-1682-5-7 (PMC3441232; doi:10.1186/2046-1682-5-7)
Supplement: Additional file 1: Supporting Information — This file contains: Additional file 1 figure S1 showing the features of the probes selected from the Nh3D data set (radius of gyration versus size), Table S1 showing the interactions between the 198 targets and the 314 random partners found in the Intact databank, Table S2 showing HEX parameters, Additional file 1 figure S2 illustrating the procedure of local patch generation for PIND computation, Additional file 1 figure S3 showing the lack of correlation between PIND and accumulated docking hits, Additional file 1 figure S4 showing the rho coefficients of Figure 2 versus standard deviation of RGEOCEN, the relative distance to the geometric protein centers, Additional file 1 figure S5, showing the multiple interfaces of the 31 proteins detected in Figure 5 and Additional file 1 Figure S6, showing the composition of surface/interface rim/interface core regions in the target data set. [file 2046-1682-5-7-S1.pdf]

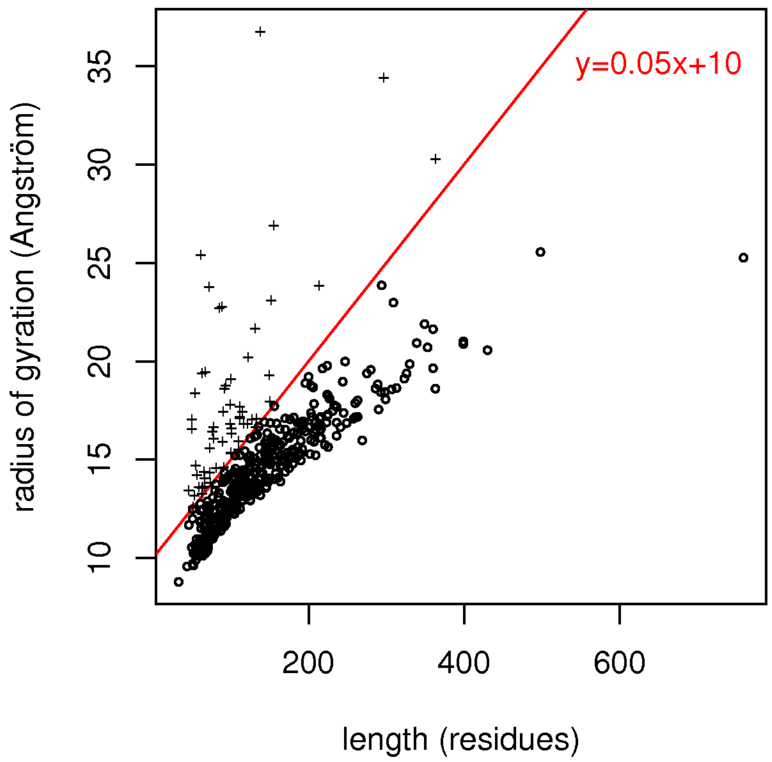

Figure S1: Length *versus* radius of gyration of structural domains from the Nh3D data set.

Circles denote compact domains and crosses denote extended domains. The limit set by y=0.05x+10 was determined empirically.

Table S1: Interactions between target proteins and compact probes detected in the IntAct database. Confidence values are in the range 0 (low confidence) to 1 (high confidence), author-score means that the confidence was assigned by the author of the publication referring to a specific interaction. Interactions with confidence ≥ 0.3 should be treated as high confidence interactions.

**Target protein (Uniprot ID): function**	**Nh3D Probe (Uniprot ID): function**	**Interaction Detection Method**	**Interaction AC number**	**Confidence Value**
1B6C_r (P62942): Peptidyl-prolyl cis-trans isomerase	1.10.750 (P40337): Von Hippel-Lindau disease tumor suppressor	anti bait coimmunoprecipitation	EBI-1059911	0.58(free-text), author-score:0.278(free-text)
1BUH_r (P24941): Cyclin-dependent kinase 2	1.10.750 (P40337): Von Hippel-Lindau disease tumor suppressor	anti bait coimmunoprecipitation	EBI-1063754	0.54(free-text) ,author-score:0.367(free-text), author-score:high(free-text)
1GHQ_r (P01024): Complement C3	1.10.750 (P40337): Von Hippel-Lindau disease tumor suppressor	anti bait coimmunoprecipitation	EBI-1069399	0.50(free-text), author-score:0.299(free-text)
1H9D_l (Q13951): Core-binding factor subunit beta	3.90.570 (P05067): Amyloid beta A4 protein	display technology	EBI-2691937	author-score:class2(free-text)
1IBR_l (Q14974): Importin subunit beta-1	2.60.210 (Q9Y4K3): TNF receptor-associated factor 6	tandem affinity purification	EBI-365110	0.49(free-text), author-score:low(free-text)
1J2J_l (Q9UJY5): ADP-ribosylation factor-binding protein GGA1	2.70.130 (P11717): Cation-independent mannose-6-phosphate receptor	far western blotting	EBI-2943702	NA
1IRA_r (P14778): ADP-ribosylation factor-binding protein GGA1	2.60.210 (Q9Y4K3): TNF receptor-associated factor 6	anti bait coimmunoprecipitation	EBI-961651	0.52(free-text)
1MQ8_l (P20701): ADP-ribosylation factor-binding protein GGA1	1.10.750 (P40337): Von Hippel-Lindau disease tumor suppressor	anti bait coimmunoprecipitation	EBI-1066494	0.55(free-text), author-score:0.153(free-text)
1NW9_r (P55211): Caspase-9	1.10.533 (O14727): Apoptotic protease-activating factor 1	x-ray crystallography	EBI-1027774	0.53(free-text)
1S1Q_r (Q99816): Tumor susceptibility gene 101 protein	1.10.540 (Q9UKU7): Isobutyryl-CoA dehydrogenase, mitochondrial	two hybrid	EBI-2339309	NA
1US7_r (P02829): ATP-dependent molecular chaperone HSP82	1.20.920 (Q03330): Histone acetyltransferase GCN5	tandem affinity purification	EBI-3811647	NA
1US7_l (Q16543): Hsp90 co-chaperone Cdc37	3.90.570 (P05067): Amyloid beta A4 protein	two hybrid array	EBI-3385594	NA
1US7_l (Q16543): Hsp90 co-chaperone Cdc37	2.60.210 (Q9Y4K3): TNF receptor-associated factor 6	anti bait coimmunoprecipitation	EBI-1062006	0.50(free-text), author-score:0.0487(free-text)
1XD3_l (P62979): Ubiquitin-40S ribosomal protein S27a	2.60.210 (Q9Y4K3): TNF receptor-associated factor 6	tandem affinity purification	EBI-365179	0.50(free-text), author-score:low(free-text)

1XD3_l (P62979):<br>Ubiquitin-40S ribosomal protein S27a	1.10.245 (Q00987):<br>E3 ubiquitin-protein ligase Mdm2	two hybrid	EBI-3932113	NA
1XD3_l (P62979):<br>Ubiquitin-40S ribosomal protein S27a	2.10.50 (P19438):<br>Tumor necrosis factor receptor superfamily member 1A	tandem affinity purification	EBI-364447	0.51(free-text), author-score:low(free-text)

Table S2. Hex parameters used in this study

receptor_range_angle	180
ligand_range_angle	180
docking_r12_range	40
docking_r12_step	0.75
docking_r12_substeps	2
max_docking_solution	3000
max_docking_clusters	2000
docking_cluster_window	200
docking_cluster_threshold	9
docking_correlation	0
docking_main_scan	18
docking_main_search	25

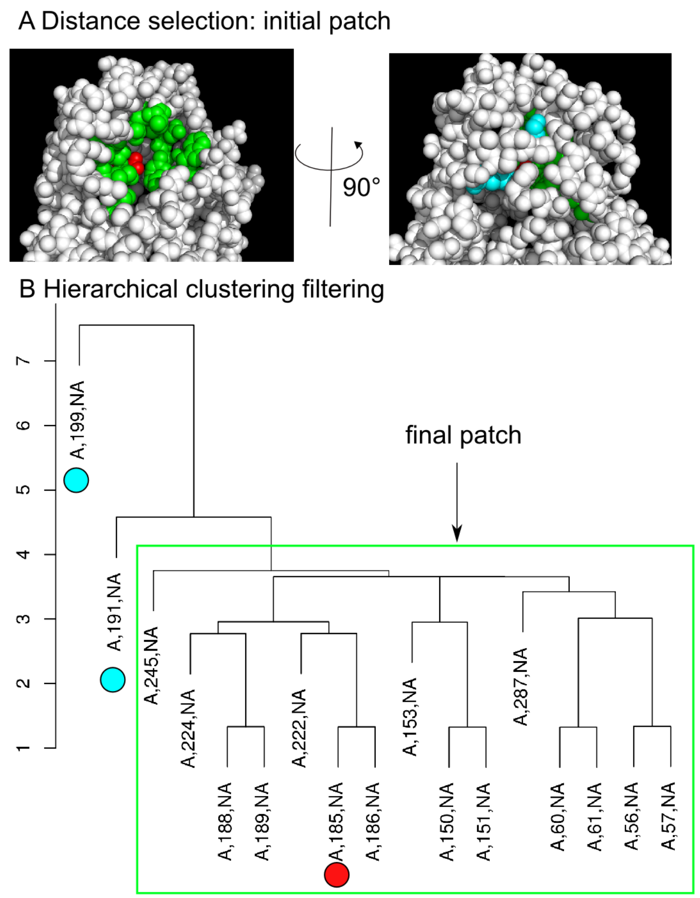

Figure S2: Example of procedure for local patch generation used in computing the planarity index P_{IND}. A local patch of exposed residues is generated around residue 185 from structure 1CLV_r. A: using a simple distance cutoff of 10Å around residue 185 (highlighted in red), neighboring residues are selected from the same face, but also from the other face of the protein (residues highlighted in green and cyan). B: filtering using hierarchical clustering (single linkage) automatically detects and discards residues from the face of the protein.

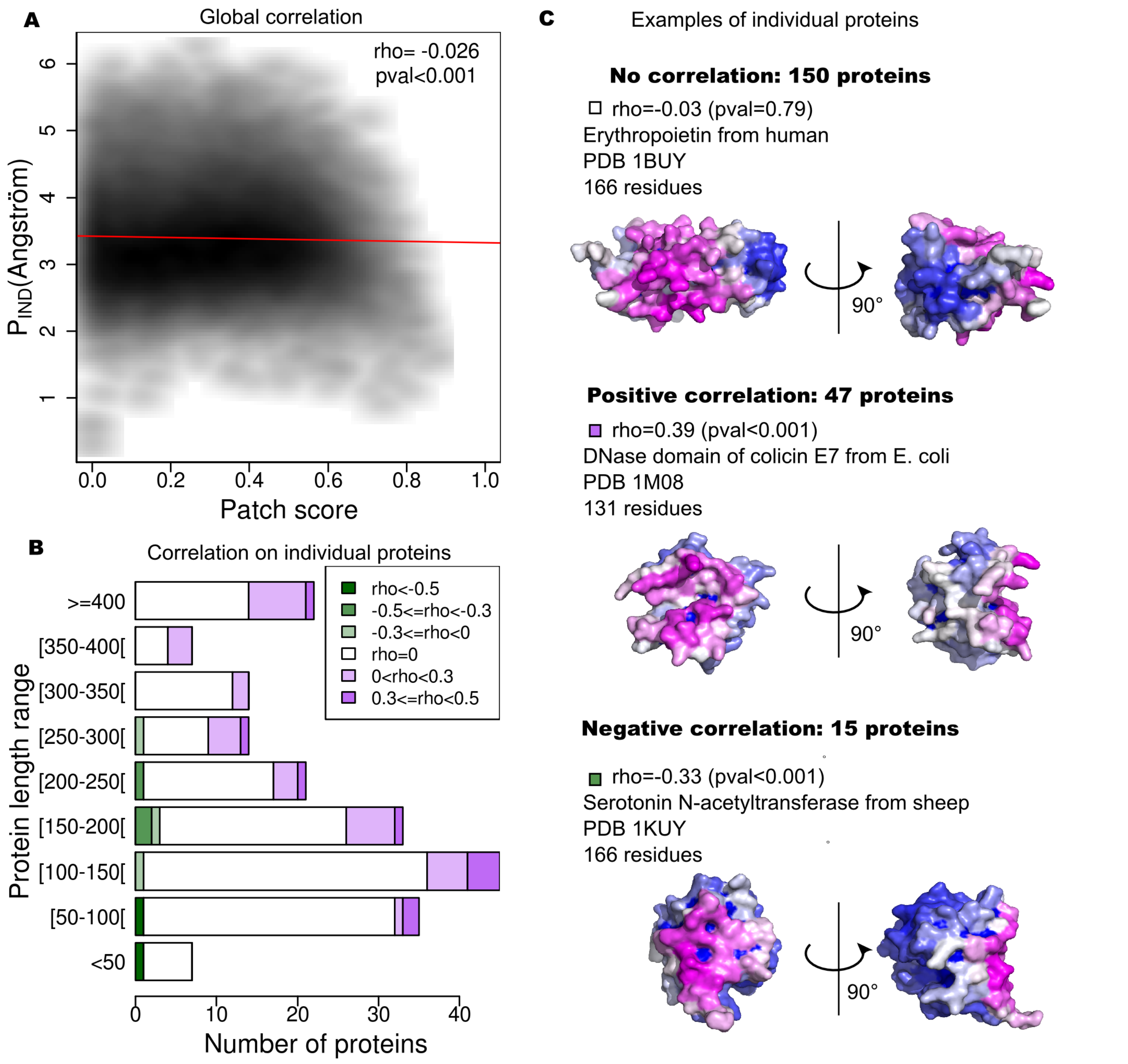

Figure S3: There is no systematic correlation between favored regions and patch planarity. A: global correlation between the planarity index, P_{IND}, and the normalized number of docking hits per patch, for all patches in the 198 proteins of the target set, after docking with the 314 compact probes. The Pearson correlation coefficient and associated empirical p-value are shown on the graph. The regression line, *y*=3.42̃0.098*x*, is shown in red. B: individual correlations for the 198 proteins of the target set. Out of 198 proteins, 150 show no significant correlation (empirical corrected p-values greater than 0.05) and 48 show a significant correlation (15 negative and 47 positive), with no evident link between rho values and protein chain lengths. If we set an arbitrary cutoff equal to 0.3 for the absolute value of rho, we obtain only 16 significant correlations (5 negatives, 11 positives). C: examples of proteins with zero, positive, and negative correlation. No correlation: 1BUY [52] (unbound form of protein 1EER_r [53]), docking hits concentrate on one side of the protein with no particular shape bias (two relatively planar areas and a protrusion). Positive correlation: 1M08 [54] (unbound form of protein 7CEI_l [41]), docking hits concentrate in a surface cleft. Negative correlation: 1KUY [55] (unbound form of protein 1IB1_l [56]), docking hits concentrate on a planar area.

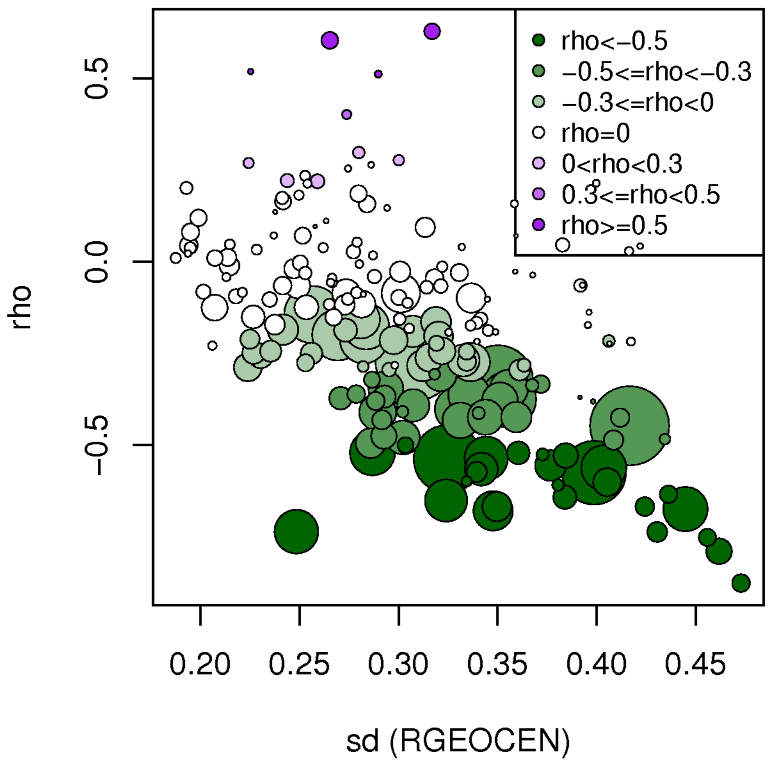

Figure S4: Link between the rho coefficients (measuring the correlation between R_{GEOCEN} and the normalized number of hits) and the standard deviation of the relative distance to the geometrical center, for the 198 proteins of the target set. There is a significant correlation between rho values and the standard deviation of relative distances, supported by a Pearson correlation coefficient equal to -0.53 (p-val=9e-16). Points are colored according to the value of rho, and their diameter is proportional to the protein size.

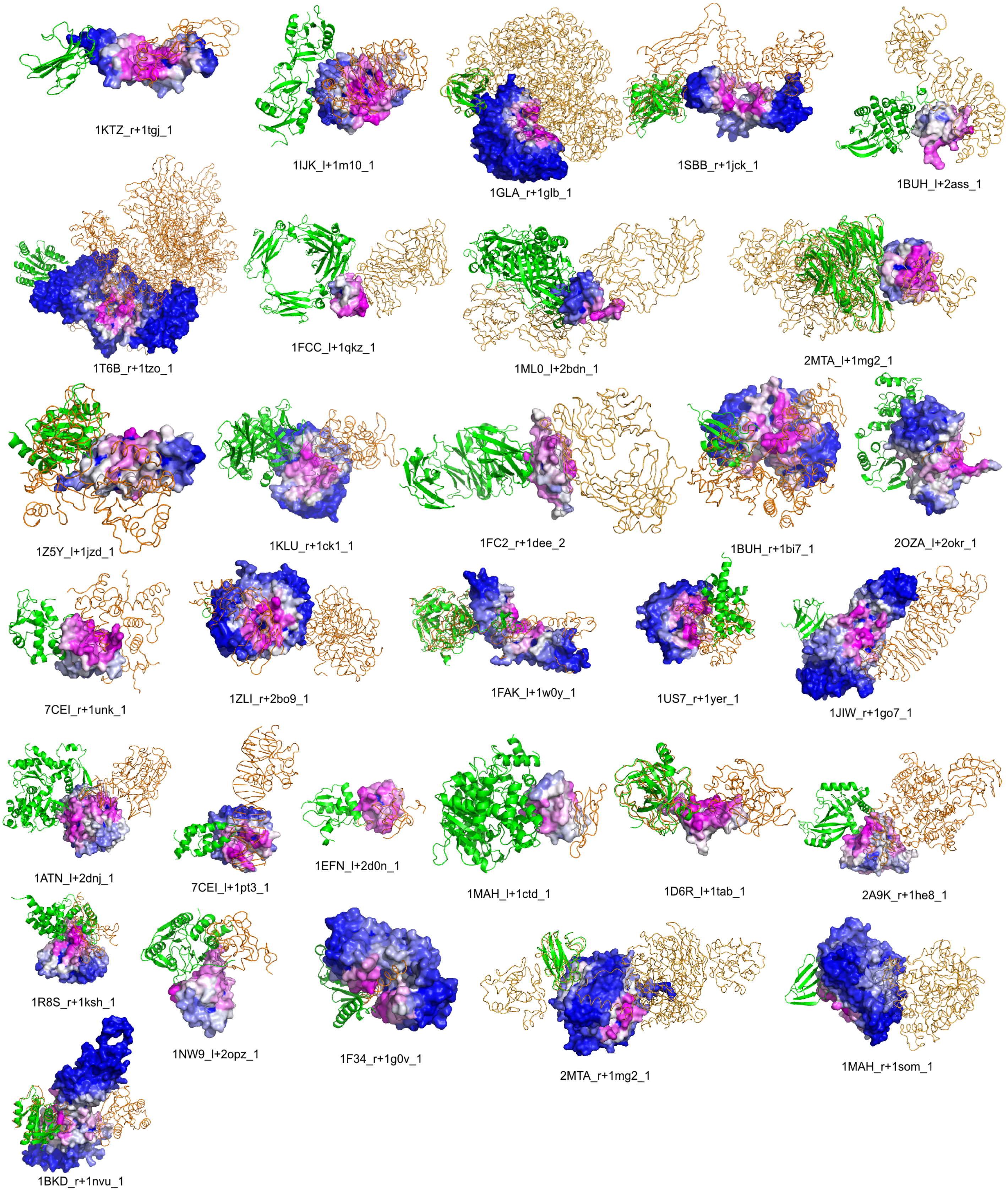

Figure S5: Visualization of the multiple interfaces for the 31 proteins extracted from the analysis in Figure 5 of the manuscript. The surface of each target protein is colored according to the number of docking hits obtained with the 25 shortest probes and using 10 models. The native partner (i.e. the one in the benchmark data set) is displayed in cartoon representation in green. The other partner identified during the manual inspection is displayed in orange. For each protein, we indicate its identifier in the benchmark data set (e.g. 1KTZ_r), the PDB code, and the assembly identifier for the structure of the alternate partner (e.g. 1tgj_1). Proteins are ordered according to the AUC values of Figure 6 of the manuscript. For protein 7CEI_l, the alternate partner is a nucleic acid.

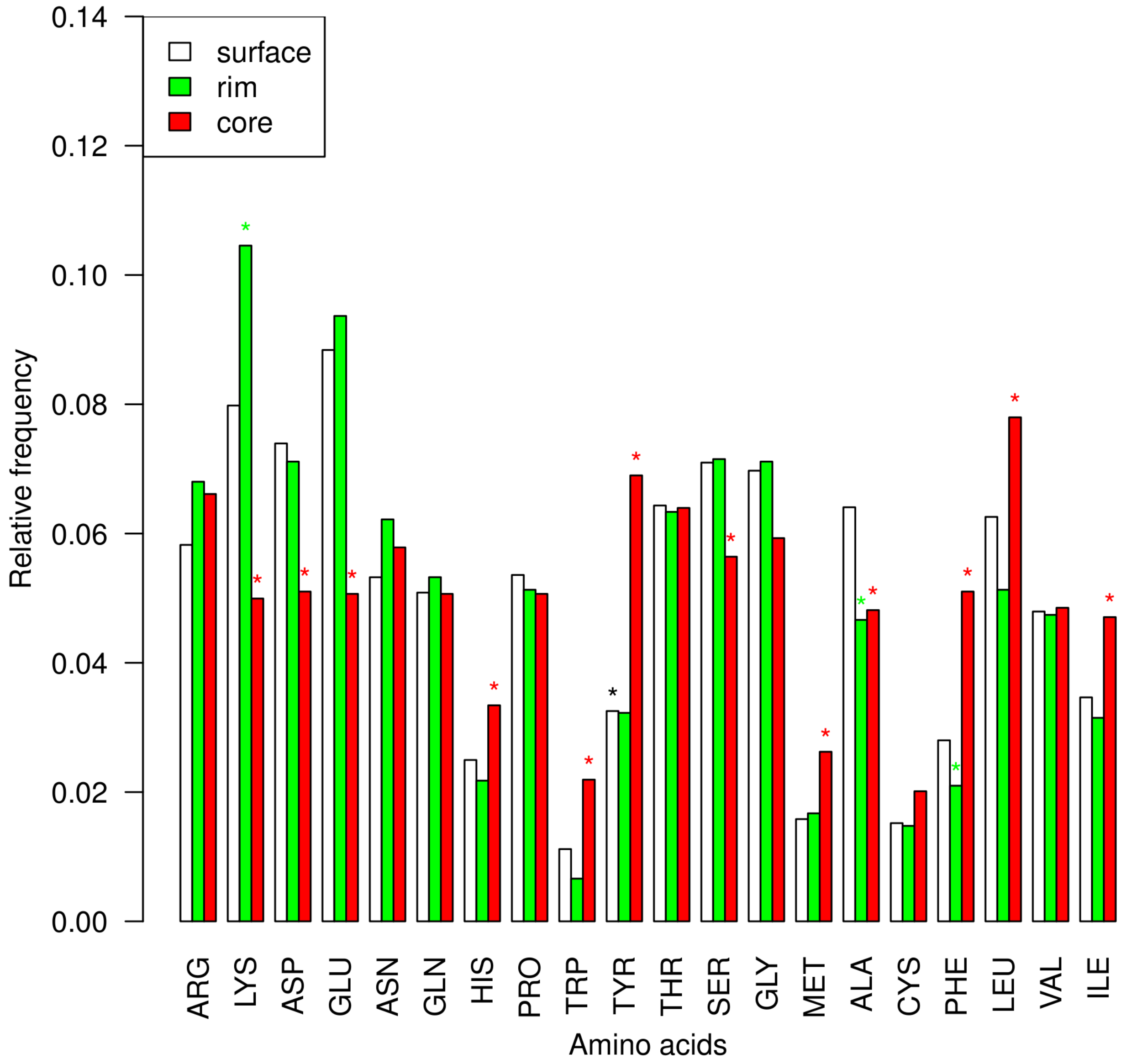

Figure S6: Composition of surface/rim/core regions in the target data set. Surface residues are defined as exposed residues (RSA>5%) not belonging to the interface. Interface residues are defined as those with a change in accessibility between the complex and the isolated form, they are further defined as core if they have at least one interface atom fully buried (ASA=0) in the complex, and otherwise as rim.
